# Immune profiling of dedifferentiated liposarcoma and identification of novel antigens for targeted immunotherapy

**DOI:** 10.1038/s41598-024-61860-3

**Published:** 2024-05-16

**Authors:** Anna Jirovec, Ashley Flaman, Elena Godbout, Daniel Serrano, Joel Werier, Bibianna Purgina, Jean-Simon Diallo

**Affiliations:** 1 Department of Biochemistry, Microbiology and Immunology, University of Ottawa, Ottawa, ON Canada; 2 Centre for Innovative Cancer Research, Centre for Cancer Therapeutics, Ottawa Hospital Research Institute, 501 Smyth Road, Box 926, Ottawa, ON K1H 8L6 Canada; 3 Department of Pathology and Laboratory Medicine, University of Ottawa, Ottawa, ON Canada; 4 Department of Pathology and Laboratory Medicine, The Ottawa Hospital, Ottawa, ON Canada; 5 Department of Orthopedic Surgery, The Ottawa Hospital, Ottawa, ON Canada

**Keywords:** Sarcoma, Tumour immunology, Cancer immunotherapy

## Abstract

Dedifferentiated liposarcoma (DDLS) is an aggressive, recurring sarcoma with limited treatments. T-cell immunotherapies selectively target malignant cells, holding promise against DDLS. The development of successful immunotherapy for DDLS requires a thorough evaluation of the tumor immune microenvironment and the identification and characterization of targetable immunogenic tumor antigens. To assess the complexity of the human DDLS tumor immune microenvironment and to identify target antigens, we used the nCounter NanoString platform, analyzing gene expression profiles across 29 DDLS and 10 healthy adipose tissue samples. Hierarchical clustering of tumors based on expression of tumor inflammation signature genes revealed two distinct groups, consisting of 15 inflamed tumors and 14 non-inflamed tumors, demonstrating tumor heterogeneity within this sarcoma subtype. Among the identified antigens, *PBK* and *TTK* exhibited substantial upregulation in mRNA expression compared to healthy adipose tissue controls, further corroborated by positive protein expression by IHC. This data shows considerable inter-tumoral heterogeneity of inflammation, which should be taken into consideration when designing an immunotherapy for DDLS, and provides a novel targetable antigen in DDLS. The results of this study lay the groundwork for the development of a novel immunotherapy for this highly aggressive sarcoma.

## Introduction

Dedifferentiated liposarcoma (DDLS) is a rare high-grade mesenchymal-derived soft-tissue malignancy that originates from adipocytes, and typically occurs within the retroperitoneal region or the extremities. 90% of DDLS tumors arise de novo and are often admixed with well-differentiated liposarcoma (WDLS) components^1^. A minority of DDLS cases arise as a recurrence of WDLS^2^. WDLS is a slow progressing low-grade sarcoma that can transition into DDLS by downregulation of adipocyte differentiation programs, resulting in tumors with a non-lipogenic DDLS adjacent to WDLS resembling mature adipose tissue. Surgical resection is the standard of care for DDLS, however tumor location, existence of metastasis, and tumor invasiveness have a significant impact on disease outcome^3^. Retroperitoneal liposarcomas are often large in size, and proximity to vital organs limits the ability to achieve negative surgical margins, leading to a 60–80% chance of local recurrence^4,5^. Additionally, DDLS has high metastatic potential, with 30% of DDLS cases metastasizing to the lungs and negatively impacting patient prognosis^6^. The resistance of DDLS to chemotherapy and radiation limits the ability to control inoperable tumors or metastatic disease^7^, resulting in 5- and 10- year survival rates of 57.2% and 40.1%^8^ and 5-year disease specific survival rates of 44%^9^. The low overall survival rate of patients with recurrent disease or metastasis highlights the necessity for the development of novel and effective therapeutic strategies against DDLS^8,9^.

As the role of the immune system in both tumor control and progression is becoming increasingly evident, modulation of the immune system has recently emerged as an alternative approach to cancer treatment^10^. Immunotherapies that have been evaluated in sarcoma include immune checkpoint inhibitors, and cancer testis antigen (CTA) targeted adoptive cellular therapies or vaccination strategies^11–17^. While CTAs are attractive targets, CTA targeted immunotherapies to date have been most effective in a small subset of sarcomas in which CTA expression is exceptionally high, such as NY-ESO-1 in synovial sarcoma^13,18,19^. Additionally, despite notable advancements in immunotherapy for various cancer subtypes, the application of immunotherapies such as immune checkpoint inhibitors (ICI) in DDLS has shown limited clinical responses. In the SARC028 study expansion cohort investigating the monoclonal antibody pembrolizumab (anti-PD1) in 40 DDLS patients, the overall response rate (ORR) stood at a mere 10%^20^. Complicating matters, sarcoma trials often lack subtype specificity—a pooled analysis of PD1/PD-L1 targeting trials in STS revealing a 7.3% ORR in DDLS patients^21^. Exploring combination regimens is an alternative approach, the combination of pembrolizumab with doxorubicin demonstrated an ORR of 35% in DDLS, however only seven DDLS patients were included in this study^22^.

The limited efficacy of ICI therapy in DDLS can be attributed in part to tumor heterogeneity and significant variations in tumor immune microenvironment^23,24^. To enhance patient outcomes, a multi-faceted approach to treatment is required. This involves the refinement of predictive biomarkers to identify patients likely to respond to therapy, along with the exploration of novel therapeutic avenues. In the most extensive study of DDLS to date, Schroeder et al*.*^25^ explored the TME, examining immune cell types, immune escape genes, and TCR sequencing in correlation with clinical outcomes. Our objective is to further advance the characterization of the TME and discern the intricate immunological and molecular mechanisms at play within the TME to guide in the development of specific and effective immunotherapies. Furthermore, CTAs remain to be promising immunotherapy targets as their expression is generally restricted to germ line cells but are aberrantly re-expressed and often up-regulated in various human cancers. However, no reliable antigen has been identified and characterized for application of antigen targeted therapies to DDLS; identifying a highly expressed tumor antigen may drive further development of novel immunotherapies for this sarcoma subtype. Alongside their role as immunotherapy targets, CTAs are currently under investigation as promising predictive biomarkers for immunotherapy response. Notably, the expression of the CTA SPA17 has been linked to tumor immune cell infiltration in a Pan-Cancer analysis, and served as a predictive indicator for patient response to anti-PD1 therapy in melanoma and urinary system tumor cohorts^26^. Similarly, in colon cancer, PBK expression has been found to correlate with heightened immune cell infiltrates, warranting additional research into PBK as a potential predictive biomarker for immunotherapy response^27^.

In this study, we performed an evaluation of the DDLS tumor immune microenvironment and cancer testis antigen expression, using RNA-based immune profiling and immunohistochemistry. Immune analysis of DDLS tumors revealed two distinct immune phenotypes; described as inflamed and non-inflamed within tumor specimens. Two novel antigens were identified that have not been previously reported as antigenic targets in DDLS.

## Materials and methods

### Ethical Statement

Archival FFPE samples were selected from patients who had provided written informed consent to The Ottawa Hospital tumor bank. This study had REB approval and was conducted under OHSN-REB Protocol #: 20,170,948-01H. All research was performed in accordance with relevant guidelines/regulations.

### Quality assessment of FFPE tissue specimens and RNA isolation

All tumor samples are post-treatment specimens obtained by surgical resection. Hematoxylin/eosin (HE) stained FFPE tumor sections were evaluated by a pathologist and selected based on quantity of DDLS and quality of tissue within section. Healthy adipose tissue controls were resected from the pannus region of DDLS patients. FFPE blocks were cut at 10 uM onto positively charged slides in RNA-free environment. Total RNA was extracted from 2 to 5 slides per case of DDLS, and 20 slides per case of healthy adipose tissue using RecoverAll™ Total Nucleic Acid Isolation Kit for FFPE (ThermoFisher Scientific, AM1975) following manufacturer’s protocol. RNA was quantified by Qubit Assay and purity was determined by Nanodrop spectrophotometer. DDLS and healthy adipose tissue RNA was diluted in RNA-free Ribo-free water at a concentration of 60 ng/ul and 20 ng/ul, respectively. Gene expression analysis was conducted by Nanostring nCounter platform using the PanCancer Immune Profiling Panel consists of 770 genes related to 14 different immune cell types, common checkpoint inhibitors, CT antigens, and genes covering both the adaptive and innate immune response. 300 ng of DDLS RNA or 100 ng of normal fat RNA in 5 uL was mixed with capture and reporter probes and hybridized for 18 h at 65 °C. Samples were scanned on a nCounter Digital Analyzer. 29 DDLS, 10 healthy adipose tissue controls were run on the nCounter Nanostring platform and passed quality control analysis performed by Nanostring Advanced analysis platform.

### Nanostring analysis

Normalization, differential gene expression analysis, and pathway analysis was performed using nSolver Advanced Analysis Software 2.0 (NanoString Technologies).

#### Differential expression analysis

For analysis of differentially expressed genes, a threshold Benajmin–Yekutieli adjusted *p*-value of < 0.005 was selected and differentially expressed genes that fell within this threshold were further analyzed. Differentially expressed genes were grouped into immune response categories determined by nSolver Advanced Analysis Software 2.0.

#### Pathway analysis

To identify active pathways within DDLS, each sample’s gene expression profile can be condensed into a set of pathway scores. Pathway scores are determined by nSolver Advanced Analysis Software 2.0, are fit using the principal component of each gene set’s data and are oriented such that increasing scores correspond to increasing expression of genes included within the pathway. Statistical analysis was performed by GraphPad Prism 6.0. Statistical significance between pathway scores of DDLS and healthy adipose controls and of expression of each individual gene within selected pathways was calculated by 2-way ANOVA tests using Sidaks multiple comparisons. Significance is based on a *p*-value < 0.05.

#### Cell type score

Immune cell profiling was performed using the genes sets on nSolver 4.0. Cell type scores are determined by Advanced Analysis Software 2.0 using expression data of genes previously shown to be characteristic of a cell population. Statistical significance of cell type scores between DDLS and healthy adipose controls was calculated by 2-way ANOVA test using Sidaks multiple comparisons using GraphPad Prism 6.0. Significance is based on a *p*-value < 0.05.

#### Immune phenotyping of DDLS

Classification of DDLS tumors into inflamed or non-inflamed phenotypes was performed based on expression of selected tumor inflammation signature (TIS) genes (*CCL5, CD27, CD274, CD8A, CMKLR1, CXCL9, CXCR6, HLA-DQA1, HLA-E, IDO1, LAG3, PDCDILG2, PSMB10, STAT1, TIGIT*) previously defined by Ayers et al.^28^ Hierarchical clustering of samples based on expression of TIS genes was performed to classify tumors and inflamed and non-inflamed^29^. Normalized mRNA counts were z-score transformed, scaled to give all genes equal variance. A heat-map was generated by hierarchical clustering using Euclidean distance and average linkage methods by nSolver 4.0.

#### Antigen expression

Antigen expression in DDLS was based on the detection of probes specific to each antigen and are considered positive when these counts are more than double the median counts of negative controls probes, as determined by Advanced Analysis Software 2.0.

### Tissue microarray (TMA) production

HE stained FFPE tumor sections were evaluated by a pathologist for adequate representation of DDLS, WDLS or normal tissue within sample, tissue quality, and level of necrosis, and appropriate tumor areas for TMA construction. The TMAs were created using samples from 72 patients, and encompassing 62 DDLS and 48 WDLS samples, and healthy matched patient tissue. Out of 72 samples 38 patients had tumors with both DDLS and WDLS components, 24 patients with only DDLS, 10 with only WDLS. Selected tissue areas were punched (1 mm needle) manually using a Veridian Tissue Arrayer and deployed into recipient paraffin wax blocks. Between 1 and 6 FFPE blocks were provided per tumor, two cores were randomly selected from each FFPE block to ensure adequate representation of whole tumor in TMA. For controls, matched normal tissue, in addition to kidney, spleen, testis and colon were included in recipient blocks. TMAs were sectioned (4um) at the Louise Pelletier Histology Core, University of Ottawa (RRID:SCR_021737).

### Immunohistochemistry

Immunohistochemical staining for CTA was performed on TMAs using MAGE-A3 (1:500, Santa Cruz Biotechnology, Dallas, TX), SSX2 (1:200, Origene, Rockville, MD), and NY- ESO-1(1:200, Santa Cruz Biotechnology, Dallas, TX), PBK (1:2000, Thermo Scientific, Walthman, MA) and TTK (1:1000, Thermo Scientific, Walthman, MA), SPA17 (1:200, Thermo Scientific, Walthman, MA). Slides were deparaffinized and rehydrated through graded CitriSolv and alcohol solutions. Antigen retrieval was performed by heat-induced epitope retrieval, in which slides were heated in sodium citrate buffer (pH 6.0) in a microwave for 10 min and cooled down to RT for 30 min. Slides were quenched in 3% H_2_O_2_ for 10 min to block endogenous peroxidase activity. After rinsing with PBS, slides were incubated for 10 min in DAKO protein block serum-free to inhibit non-specific staining. Slides were rinsed in PBS and treated with primary antibodies at 4 °C overnight. ImmPRESS® HRP Anti-Mouse IgG (Peroxidase) Polymer Detection Kit is applied to slides and incubated for 30 min at room temperature. Staining was visualized with DAB (5-min development). Slides were counterstained in Harris Modified hematoxylin and dehydrated through graded ethanol and CitriSolv solutions. Testis tissue was used as a positive control for all cancer testes antigens staining.

### IHC scoring and statistical analysis

Immunohistochemical staining was graded by a pathologist. The staining intensity is graded as 0 (no staining), 1 (weak), 2 (moderate), or 3 (strong). The percentage of positive cells is graded as 0 (negative), 1 (1–25%), 2 (26–50%), 3 (51–75%), or 4 (76–100%). The IHC score is calculated by multiplication of staining intensity and percentage of positive cells. The final IHC score is the mean of values recorded for each individual case. Statistical analysis was performed by GraphPad Prism 6.0. Statistical significance of antigen expression between DDLS and WDLS was calculated by 2-way ANOVA tests using Sidaks multiple comparisons. Significance is based on a *p*-value < 0.05.

## Results

### Patient characteristics

Archival tumor samples were used to determine the immune profile and to identify antigens expressed in DDLS using RNA-based tumor profiling. RNA was extracted from formalin-fixed paraffin-embedded (FFPE) DDLS tumor samples selected from 29 patients (Table 1, Supplemental Table 1), and matched with healthy adipose tissue from the pannus fat of 10 patients. The selection included 24 primary tumors (82.8%) and 4 recurrent tumors (13.7%), with the location of one tumor unavailable. DDLS tumors occurred in the retroperitoneum in 9 patients (31%), trunk in 4 patients (13.7%) and 14 in extremities (48.3%). 8 patients in the study were only treated by surgical resection. 5 (17.2%) patients received neoadjuvant radiation therapy, 6 (20.7%) patients received adjuvant radiation therapy, and 1 (3.4%) patient received adjuvant chemotherapy and radiation therapy. Tumor samples from patients that received neoadjuvant treatment are post-treatment surgical specimens. Out of 29 patients, 11 patients (37.9%) were alive at the time of last follow-up.

**Table 1:** Summary of patient characteristics of FFPE DDLS samples used in nCounter Nanostring analysis.

Characteristics	N = 29	%
Age		
Median	66.5	
Range	31–88	
Site category		
Retroperitoneal	9	31
Trunk	4	13.7
Extremity	14	48.3
Other	1	3.4
NA	1	3.4
Primary or recurrent tumors		
Primary tumor	24	82.8
Metastasis present	5	20.8
No metastasis present	10	41.7
NA	9	37.5
Recurrence	4	13.7
Metastasis present	0	0
No metastasis present	1	25
NA	3	75
Unknown	1	3.4
Treatment		
Surgery alone	8	27.5
Neoadjuvant therapy		
RTX	5	17.2
Adjuvant therapy		
RTX	6	20.7
CTX + RTX	1	3.4
Treatment unknown	9	31
Outcome		
Disease-free at time of last follow-up	9	31
Lost to follow-up & NA	15	51.7
Deceased from disease	4	13.7
Deceased not from disease	1	3.4

### Differential gene expression analysis revealed upregulation of genes associated with immune functions

**Figure 1:**
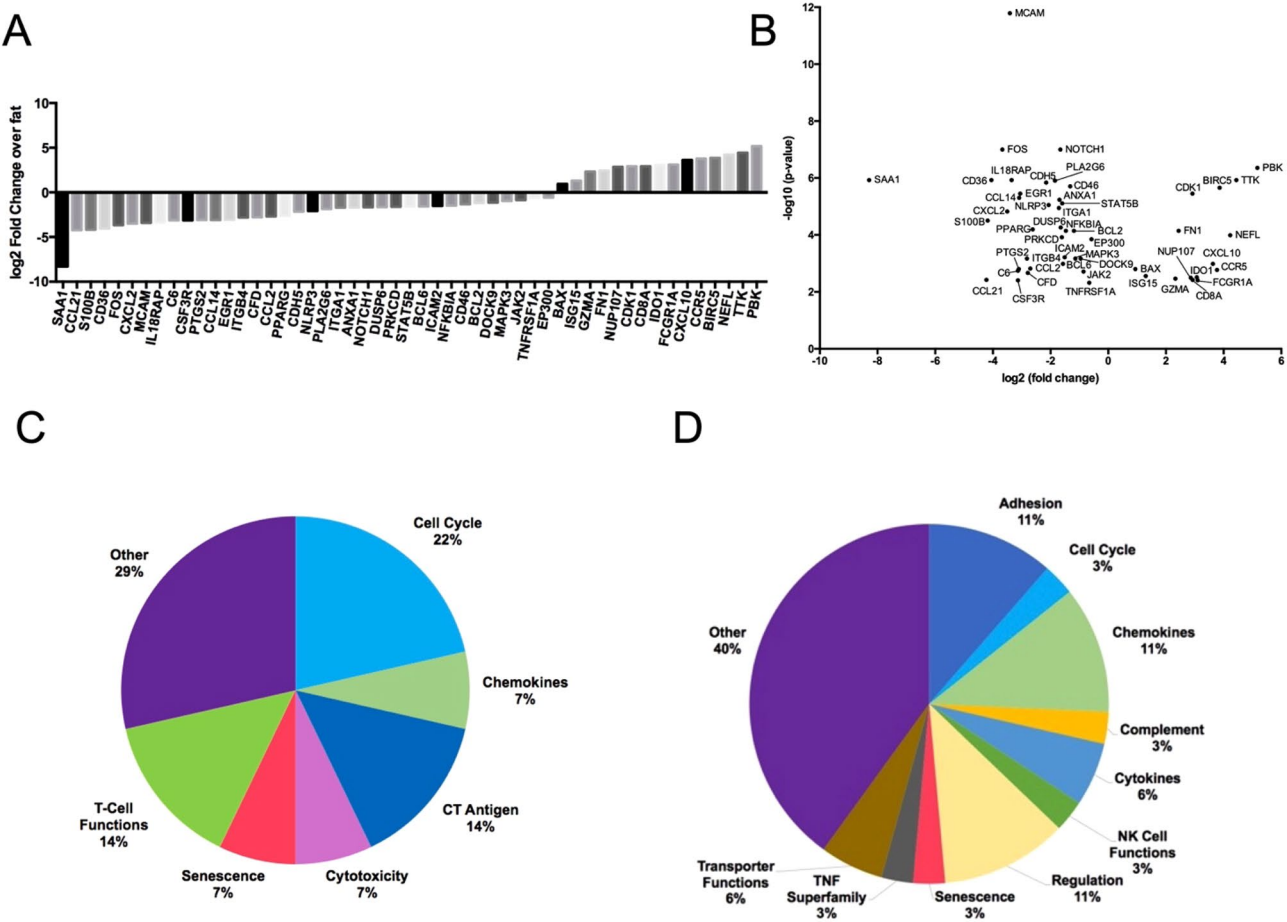
Distinct expression pattern characteristics of DDLS tumors compared to healthy adipose tissue. (**A**) Genes showing significant (*p*-value < 0.005) fold change in expression compared to control tissue. Significance calculated using the Benjamini-Yekutieli procedure (**B**) Volcano plot showing statistical significance (− log10 *p*-value) versus fold change of differentially expressed genes. Overview of gene sets encompassing differentially expressed genes in DDLS compared to healthy adipose controls that are (**C**) upregulated and (**D**) downregulated.

To identify the key differences in gene expression between DDLS tumors and healthy adipose controls, mRNA expression of 770 genes was determined using the nCounter PanCancer Immune Profiling Panel on the nCounter SPRINT Profiler. Differential gene expression analysis was performed and a p-value < 0.005 threshold was set for significant differences in gene expression. 51 genes that fall within this threshold were further analyzed and classified as genes involved in cytokine signaling, cell cycle, T cell function, adhesion and CTAs. (Supplemental Table 2, Fig. 1A–D).

Genes that were upregulated in DDLS compared to healthy adipose controls include cytokines (*CXCL10, CCR5*), genes involved in cell cycle (*CDK1, BIRC5, BAX*), T cell functions (*GZMA, CD8, IDO1*) and CT antigens (*TTK, PBK*) and others such as *ISG15, FN1, NEFL* and *NUP107* and *FCGR1A* (Fig. 1A,C). Together, upregulation of these genes is indicative of a tumor undergoing proliferation that is permissive to immune cell infiltration.

Genes that are downregulated in DDLS compared to healthy adipose controls include adhesion molecules *ITGA1, ITGB4, ICAM2* and *MCAM*. There is also downregulation of several cytokines and chemokines and their receptors, such as *IL-1b, CCL21, IL18RAP, TNFRSF1A*, and several members of the JAK/STAT pathway (*JAK2*, *NFKB1A* and *STAT5B*). Several downregulated genes are associated with loss of adipocyte function and include *SAA1*, *PPARG*, and *DUSP6* and the cytokines *CCL14*, *CXCL2* and *CCL2*. (Fig. 1A).

### DDLS show heterogeneity in active pathways and infiltrating lymphocytes

To obtain information about the specific underlying molecular changes in DDLS, we assessed active molecular pathways. In this approach, the expression data of all genes in the nCounter panel was subjected to a pathway analysis performed by nSolver 4.0. Pathway scores are determined by nSolver Advanced Analysis Software 2.0 and are fit using the principal component of each gene set’s data and are oriented such that increasing scores correspond to increasing expression of genes included within the pathway. Pathway scores were compared between DDLS and healthy adipose controls. We observed wide distribution of pathway scores in DDLS samples compared to healthy adipose controls (including adhesion, antigen processing, B-cell functions, cell cycle, cell functions, chemokines, complement, cytotoxicity, macrophage functions, NK cell functions, regulation, T-cell functions, TNF superfamily, and transporter functions). This suggests heterogeneity in active molecular pathways between DDLS samples.

**Figure 2:**
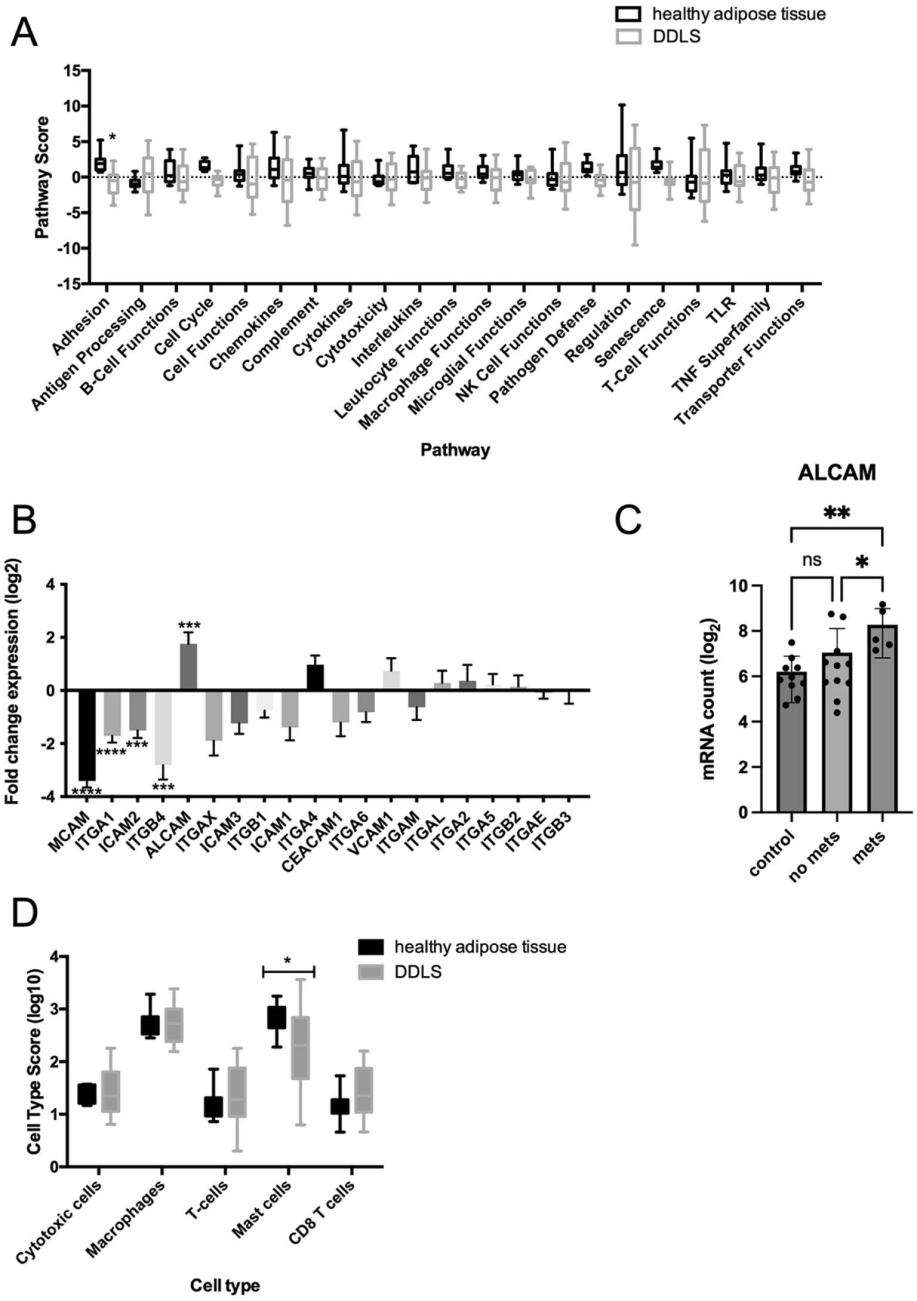
Active molecular pathways and cell types within DDLS reveal tumor heterogeneity. (**A**) Overview of pathway scores in DDLS compared to healthy adipose tissue. Significance calculated using 2way ANOVA Sidak’s multiple comparisons test. * = *p*-value < 0.05, ** = *p*-value < 0.01 (**B**) Bar graphs showing log2 fold change in expression of individual genes within the adhesion pathway. Significance calculated using the Benjamini-Yekutieli procedure. * = *p*-value < 0.05, ** = *p*-value < 0.01, *** = *p*-value < 0.001, **** = *p*-value < 0.0001 (**C**) log2 mRNA counts of *ALCAM* in healthy adipose tissue, primary tumors of patients with metastasis, and those without metastasis. Significance calculated using One way Anova, * = *p*-value < 0.05, ** = *p*-value < 0.01 (**D**) Cell type scores of different immune cells detected in DDLS.

The only pathway significantly downregulated in DDLS compared to healthy adipose controls was cell adhesion. (Fig. 2A). Within the adhesion pathway, *MCAM, ITGA1, ICAM2* and *ITGB4* demonstrate significant downregulation in DDLS compared to healthy adipose tissue. (Fig. 2B). The activated leukocyte cell adhesion molecule (*ALCAM*) was the sole gene that demonstrated significant upregulation (*p* < 0.005) in DDLS when compared to healthy adipose tissue. (Fig. 2B) Cell adhesion molecules such as integrins and members of the immunoglobulin superfamily *(MCAM, ALCAM)* have been associated with metastatic behaviour^30–32^. Therefore, we explored the differential expression of adhesion molecules between primary tumors of patients with or without metastasis and observed significantly increased expression of *ALCAM* in the primary tumors of patients with metastasis (Fig. 2C).

Next, we assessed immune cell types present in DDLS samples compared to healthy adipose controls. Cell type scores were calculated by nSolver Advanced Analysis Software 2.0 based on the expression levels of predefined genes previously shown to be characteristic of a cell population. Cell types that were detected by nCounter Nanostring are classified by nSolver as cytotoxic cells, DC, macrophages, T-cells, mast cells and CD8 T cells. Cytotoxic cells, DCs, macrophages, T-cells and CD8 T cells showed no significant differences in abundance compared to healthy adipose controls (Fig. 2D). The increase of mast cells in healthy adipose controls compared to tumor tissue aligns with the dedifferentiation of adipose tissue observed in progression to DDLS as mast cells play an active role in adipose tissue development and metabolism^33,34^. Notably, we observed broader distribution of cell type scores of all tumor-infiltrating cell types identified in DDLS compared to healthy adipose tissue. This observed difference suggests inter-tumoral heterogeneity in immune cell burden within DDLS.

### Immune profiling reveals DDLS tumors have two distinct immune phenotypes

**Figure 3:**
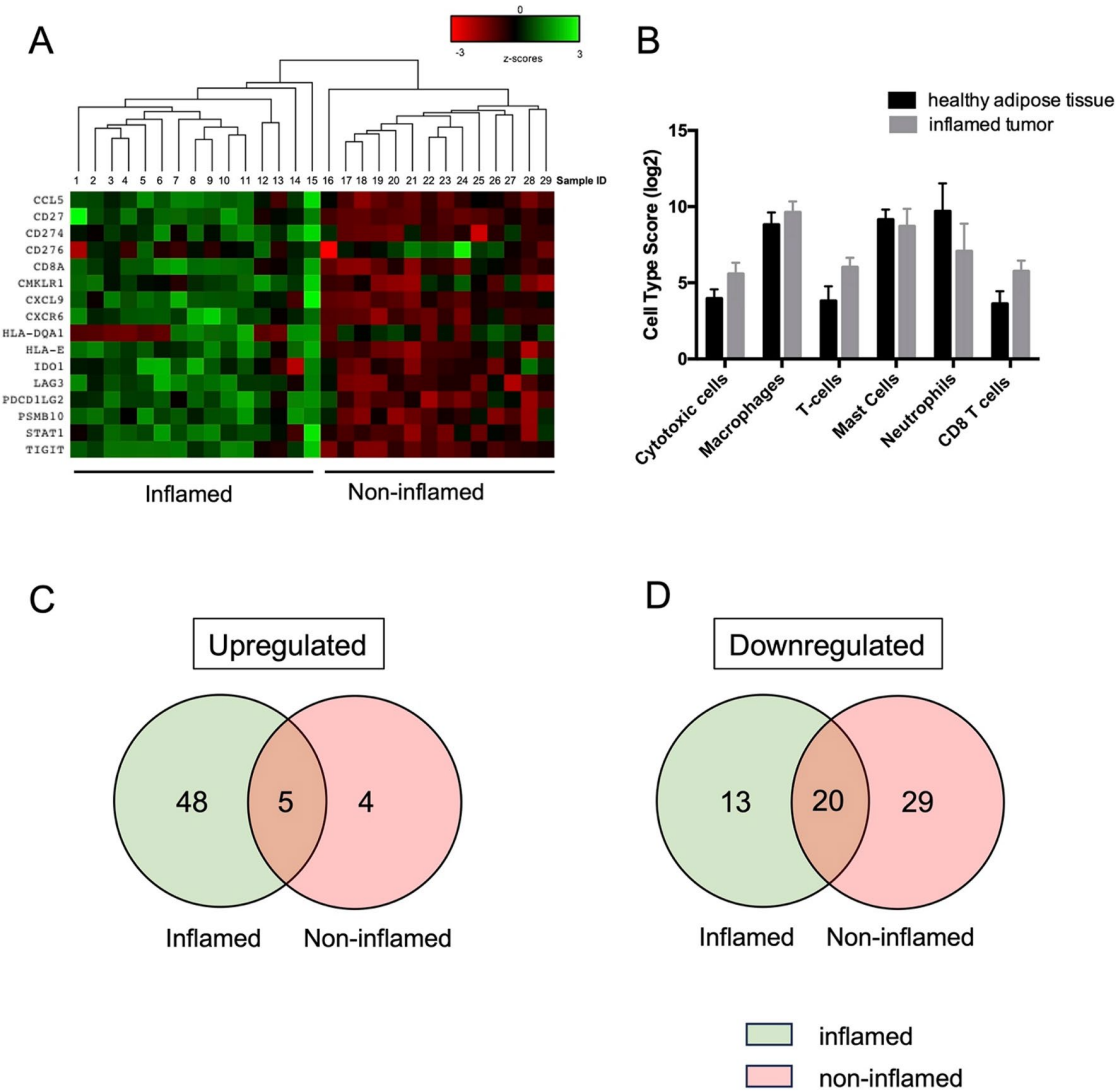
Immune profiling of DDLS reveals two distinct tumor immune microenvironments (**A**) Heatmap of mRNA normalized counts of tumor immune-related genes in DDLS tumors. Data was z-score transformed, scaled to give all gene equal variance, and heat-map generated by hierarchical clustering using Euclidean distance and average linkage method. Clustering reveals two inflammation signatures, separating samples into 15 inflamed and 14 non-inflamed tumors. (**B**) Cell type scores of immune cells detected in inflamed DDLS tumor. No statistically significant differences were identified. Venn diagrams showing overlap of significantly differentially expressed genes (**C**) upregulated and (**D**) downregulated by inflamed and non-inflamed tumors. Significance calculated using the Benjamini-Yekutieli procedure, a *p*-value < 0.005 threshold was set for significant differences in gene expression.

The observed heterogeneity in pathway scores and the abundance of various immune cell types in DDLS compared to healthy adipose controls prompted us to question whether DDLS tumors can be subdivided based on expression of inflammatory genes. To evaluate the immunological characteristics of DDLS tumors, we classified them using 16 genes from the Tumor Inflammation Signature (TIS). This signature includes a distinct set of IFNγ-signaling related genes, and can be used to distinguish tumors with pre-existing inflammatory components and non-inflamed tumors^35^. The TIS contains genes related to antigen presentation, chemokine expression, cytotoxic activity, and adaptive immune resistance. Hierarchical clustering based on expression levels of TIS genes revealed two distinct groups, consisting of 15 inflamed tumors and 14 non-inflamed tumors, demonstrating a dichotomy within the DDLS sarcoma subtype (Fig. 3A, Supplemental Table 3). To validate the categorization of these tumors into the two distinct phenotypes, we conducted expression analysis of the immune cell marker *CD45* between the newly classified groups and found significant upregulation of *CD45* expression in the inflamed group, confirming the accurate classification of tumors (Supplemental Fig. 1A).

The analysis above confirms at least two distinct immune-inflammatory phenotypes in DDLS tumors, therefor subsequent immune and expression analyses were conducted separately for each phenotype. Cell types were classified as described above. In inflamed tumors, cell types that were detected at statistically significant levels (p-value < 0.05) include cytotoxic cells (*GZMB, PRF1, GZMH, KLRB1, GZMA, CTSW*), macrophages (*CD84, CD163, CD68*), T cells (*SH2D1A, CD3D, CD3E*), mast cells (*TPSAB1, MS4A2*), neutrophils (*CSF3R, S100A12*) and CD8 T cells (*CD8A, CD8B*) (Supplemental Fig. 1B). Cytotoxic cells, macrophages, T cells and CD8 T cell scores were higher in inflamed tumors compared to healthy adipose controls, however, did not reach statistical significance (Fig. 3B). In non-inflamed tumors, only dendritic cells were detected (Supplemental Fig. 1C).

Differential expression analysis of inflamed and non-inflamed tumors compared to healthy adipose tissue revealed shared genes in both upregulated and downregulated immune-inflamed profiles, as shown in Fig. 3C,D. Five significantly upregulated genes are shared between both immune phenotypes; *FN1, CDK1, BIRC5, TTK* and *PBK* (Fig. 3C). In contrast, twenty downregulated genes were shared between the two immune sub-types of DDLS and are involved in adhesion and loss of adipocyte function (Fig. 3D).

Altogether, this data shows that DDLS can have two distinct immune phenotypes that demonstrate different levels of immune cell infiltration and expression of immune related genes.

### Cancer testis antigens are transcriptionally expressed in DDLS

**Figure 4:**
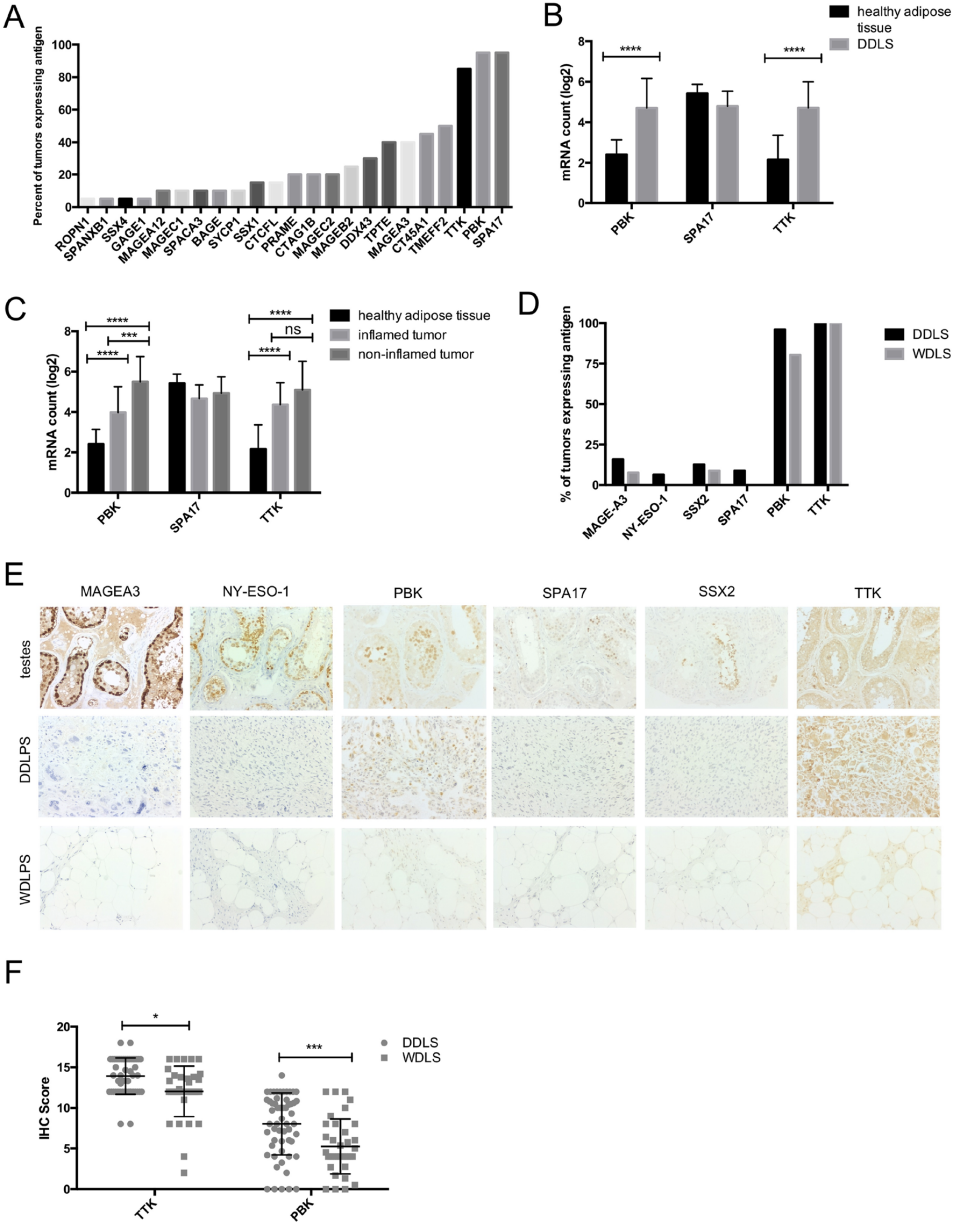
Analysis of mRNA and protein expression of cancer-testis antigens in liposarcoma allows for identification of targetable antigens (**A**) Percentage of tumors expressing CTA mRNA (**B**) mRNA expression of antigens in healthy adipose tissue and DDLS (**C**) mRNA expression patterns of *TTK*, *PBK* and *SPA17* in healthy adipose tissue, inflamed and non-inflamed tumors. (**D**) Percent of tumor specimens expressing CTAs determined by IHC. (**E**) Representative staining of human testis (positive control), DDLS and WDLS for CTAs MAGE-A3, NY-ESO-1, PBK and SSX2. (**F**) IHC scores of PBK and TTK expression, IHC score is determined by staining intensity and percentage of positive cells. Significance calculated using 2way ANOVA Sidak’s multiple comparisons test. **** = *p*-value < 0.0001, *** = *p*-value < 0.001.

The development of targeted immunotherapy for DDLS relies on the identification and characterization of targetable tumor antigens. The 770 gene Nanostring Immune Profiling Panel includes probes to detect mRNA gene expression of 30 CTAs. Out of 30 CTAs included, 23 were expressed at the mRNA level in DDLS (Fig. 4A). From the results in Fig. 4A, it emerges that the three most frequently observed antigens are TTK protein kinase (TTK), Lymphokine-activated killer T-cell-originated protein kinase (PBK) and Sperm Autoantigenic Protein 17 (SPA17), detected in over 80% of DDLS tumors. Looking further at average mRNA expression levels for these antigens reveals that *TTK, PBK* and *SPA17* are the most highly expressed in DDLS compared to other CTAs (Supplemental Fig. 2A). These antigens were therefore selected for further analysis and validation. We next compared the mRNA expression of these antigens in DDLS relative to healthy adipose tissue, and *PBK* and *TTK* showed a significant increase in mRNA expression (Fig. 4B). Interestingly, we found that *TTK* and *PBK* were often co-expressed and observed a weak (r^2^ = 0.4547) but highly statistically significant (p-value < 0.0001) correlation between their expression levels (Supplemental Fig. 2B). While *SPA17* was expressed at the highest levels in DDLS, it was also expressed at high levels in healthy adipose controls.

We further sought to delineate *TTK* and *PBK* expression in DDLS tumors across inflamed and non-inflamed classifications as outlined in Fig. 3A. Both inflamed and non-inflamed tumors showed significant increases in expression of *TTK* and *PBK* compared to healthy adipose tissue controls (Fig. 4C). Consistent with Fig. 4B, *SPA17* expression was not significantly different compared to healthy adipose controls in both inflamed and non-inflamed tumors (Fig. 4C).

Overall, analysis of mRNA expression of 30 CTAs revealed three candidate antigens, *TTK*, *PBK* and *SPA17* that are expressed in the majority of inflamed and non-inflamed DDLS tumors, however, only TTK and PBK demonstrate increased expression compared to healthy adipose tissue controls.

### Cancer testis antigens proteins are expressed in DDLS and WDLS

T-cell based immunotherapies targeting antigens exert their anti-tumor function following antigen specific T-cell engagement with MHC-antigen complexes presented at the surface of tumor cells. Antigens must be expressed at the protein level to be loaded onto MHC molecules, therefore, analysis of protein expression of potential target antigens in tumors is critical. Furthermore, DDLS often present as a biphasic neoplasm containing WDLS components^3^, therefore, it is important to confirm antigen expression in both WDLS and DDLS. We performed immunohistochemistry (IHC) on DDLS and WDLS to confirm protein expression of TTK, PBK and SPA17, as well as CTAs commonly expressed in sarcoma including NY-ESO-1 (included in Nanostring panel as *CTAG1B*), SSX2 and MAGE-A3, some of which that have been targeted by adoptive cell therapy^18,36^.

IHC staining was observed with varying frequency for all antigens in both WDLS and DDLS (Fig. 4D,E). However, TTK and PBK were the most frequently expressed (Fig. 4D). TTK was expressed in 100% of DDLS (52/52) and WDLS (36/36) samples. PBK was found to be expressed in 96% (52/54) of DDLS samples and 80.4% (33/41) of WDLS samples. (Fig. 4D). The next most expressed antigen was MAGE-A3 which was observed in 15.87% (10/63) of DDLS and 7.67% (3/45); followed by SSX2 in 12.7% (8/63) of DDLS and 8.88% (4/45) of WDLS; then NY-ESO-1 in 6.35% (4/62) of DDLS and not detected in WDLS. Finally, SPA17 was detected only in 5.5% (3/54) of DDLS, in sharp contrast with transcriptional data (Fig. 4A) and was not detected in WDLS. Overall IHC scoring intensity for the two most frequently observed antigens showed significantly higher expression of both TTK and PBK in DDLS compared to WDLS (Fig. 4F). Staining of healthy tissue for PBK and TTK revealed PBK expression in testes and Paneth cells in the colon (Fig. 4E, Supplemental Fig. 3A), contrastingly, TTK was expressed in all normal tissues tested (Supplemental Fig. 3A, B).

In this study, it is important to note that DDLS samples were not uniformly represented on the TMA, with the number of cores per patient ranging from 2 to 12. To assess whether this variability in sampling could introduce bias, specifically whether we are detecting more antigen expression in DDLS tumors with more sampled cores from heterogenous regions, we analysed the correlation between IHC scores and the number of cores assessed. This analysis of PBK staining revealed no significant association between these parameters (Supplemental Fig. 4).

## Discussion

DDLS is a rare sarcoma subtype with poor prognosis and limited novel therapeutics in clinical development. Cancer immunotherapy has emerged as an alternative to conventional treatment options and employs different platforms to stimulate the immune system to reject tumors. A strong understanding of the immune mechanisms at play within the tumor and the characterization of targets are essential for the design of successful immunotherapy. However, current knowledge of the immune profile and targetable antigens in DDLS is limited. Immunological investigations thus far include small sample sizes or have predominantly centered on soft-tissue sarcomas as a whole, lacking in-depth studies dedicated to DDLS^37,38^. Furthermore, no consistent and reliable tumor antigen has been identified in DDLS.

The goal of this study was to investigate the expression of immune-related genes and identify CTAs unique to DDLS that would direct future studies on effective immunotherapeutic treatment for this sarcoma subtype. Analysis of expression patterns characteristic of DDLS tumors was performed leveraging the nCounter Nanostring platform. We assessed differentially expressed genes between DDLS tumors and healthy adipose tissue, identified distinct inflamed and non-inflamed profiles for DDLS, and identified a potentially promising antigen to drive future development of target immunotherapies for DDLS.

Immune cell profiling of DDLS tumors compared to healthy adipose tissue revealed large tumor heterogeneity in the quantity and type of infiltrating leukocytes, as previously described^39^. Clustering of DDLS samples based on expression levels of selected Tumor Inflammation Signature genes revealed two distinct immune phenotypes; termed inflamed and non-inflamed, with DDLS tumors being approximately distributed equally between both subtypes. In our study, inflamed tumors demonstrate increased levels of cytotoxic cells, macrophages, T-cells, mast cells, neutrophils and CD8 T cells. Importantly, T-cells present within the TME suggest that DDLS may be permissive to T cell infiltration upon generation of a large pool tumor-specific T cells after administration of an immunotherapy.

Several studies have explored the immune classification of STS, encompassing DDLS. Weng et al*.*^40^ identified three immune groups in STS, including “hot” and “cold” immune groups. Classification of two sarcoma cohorts (TCGA and GSE21050) into immune groups revealed contrasting distribution of DDLS in “hot” vs “cold” between the two datasets. This difference underscores the variability in immune subtype distribution across different datasets, as well as heterogeneity within the DDLS subtype. Similarly, Petitprez et al*.*^24^ conducted gene expression analysis across various STS, which resulted in an immune-based classification featuring five distinct immune subtypes. Interestingly, DDLS exhibited distribution across all identified groups, highlighting its inherent heterogeneity. However, due to the different approaches for immune classification in these studies, it remains challenging to draw direct correlations with our data. Moreover, our sample set under-represents retroperitoneal DDLS, primarily focusing on extremity DDLS. It is important to note that the predominant sites for DDLS are the retroperitoneum, followed by the extremities, trunk, and head and neck^41^. This under-representation introduces an additional confounding factor.

While certain sarcoma subtypes, such as ASPS and UPS, demonstrate a high likelihood of response to immunotherapy^42–44^, the immunophenotype of STS can also impact clinical outcome, as revealed by several studies, including in DDLS^24,25,35^. Classification of tumors in our study relies on genes related to IFNγ signalling, which includes genes featured in the Tumor Inflammation Signature score – an algorithm designed for predicting patient response to pembrolizumab. While we did not employ the algorithm itself in our study, the approximate 50/50 distribution of DDLS tumors between inflamed and non-inflamed phenotypes implies that around half of DDLS patients could potentially respond favorably to ICI. Although this score has not been validated specifically in sarcoma, its efficacy in predicting responders to pembrolizumab has been demonstrated in retrospective analysis in melanoma and NSCLC^45^. Our study was not designed to identify clinical correlates, focusing instead on understanding the TME, however future investigations should explore the potential application of TIS score in clinical trials focusing on pembrolizumab, where current selection criteria include patients without biomarker selection, other than PD-L1 expression. Consistent with this idea, Petitprez et al*.*^24^ revealed that tumors with high immune infiltrate and tertiary lymphoid structures (TLS) showed the best response to pembrolizumab, introducing TLS as a potential biomarker. As a result, the PEMBROSAC study introduced a new cohort of patients with TLS to investigate the efficacy of pembrolizumab in tumor with TLS. This study confirmed that patients with TLS demonstrate a better response, highlighting the need for immune-based stratification in future STS studies^46^. In our study, inflamed tumors demonstrated high expression of CXCL13, a common TLS marker, suggesting responsiveness to immune checkpoint inhibitors.

Immunotherapy for DDLS has been predominantly centered around ICI’s. However, numerous studies focusing solely on checkpoint inhibitors have consistently reported limited efficacy in this subtype, indicating that checkpoint inhibition alone may not merit further exploration in unselected sarcoma cohorts. The shift towards combination therapies is gaining traction, and the challenge lies in determining the most effective combinations, which may vary across different sarcoma subtypes. Recent clinical studies have demonstrated that overall response rates (ORR) can be enhanced through combinatorial approaches involving ICIs and systemic therapy^47^.

Based on the findings from our study, IDO1 emerges as a potential therapeutic target in DDLS. We observed significant upregulation of *IDO1* in DDLS. IDO1 is induced by pro-inflammatory cytokines and controls immune responses by conversion of local tryptophan to the immunosuppressive metabolite kynurenine^48^. Given the biological importance of IDO1 in cancer immune escape, several IDO1 inhibitors have advanced into clinical trials as monotherapies or in combination with conventional therapies for cancer treatment^49^. A Phase II study of an IDO1 inhibitor (epacadostat) in combination with pembrolizumab for treatment of advanced metastatic sarcoma (NCT03414229) has been conducted, although the results did not show optimal outcomes, there was no inclusion of DDLS in this study^50^. Despite the results of this trial, the success of this combination in other cancers such as melanoma suggests this combination is worth exploring, especially given the limited therapeutic options available for DDLS^51,52^. Our study, and others provide, rationale for further exploration and consideration of IDO1 as a viable target in DDLS^53^.

It is crucial to extend our exploration beyond checkpoint inhibitors to encompass a broader spectrum of potential therapeutic avenues for DDLS. Obvious considerations lie in CDK4 and MDM2 inhibitors, as they target the main driver proteins of DDLS. Exploring additional targets, such as those related to metastasis, could offer valuable insights into novel treatment strategies for DDLS. We observed significantly increased expression of *ALCAM* in tumors from patients with metastasis present compared to those without metastasis. These findings offer valuable insights into the potential role of *ALCAM* as a contributing factor to metastasis in DDLS, and suggest its usefulness as a target^54^. Lastly, further exploration into alternative immunotherapies is essential. For example, the combination of the oncolytic virus (OV) T-VEC (talimogene laherparepvec) with pembrolizumab has shown promise in certain contexts and warrants further investigation as a potential therapeutic approach for DDLS^55^. In the context of the immune classification in our study, it is crucial to adopt strategies effective for “non-inflamed” tumors than can transform the TME into a more immunogenic state – which OVs can achieve. Notably, our group has shown that sarcomas are highly sensitive to the oncolytic effects of a panel of oncolytic virus platforms^56^.

This study focused on characterizing CTAs, a class of antigens considered to be promising immunotherapeutic targets due to their restricted expression to germline cells, overexpression in cancer, and their immunogenic nature. CTAs that have emerged as target candidates include MAGE-A3, NY-ESO-1 and SSX2, with several clinical trials evaluating immunotherapies targeting these antigens underway or completed (NCT02111850, NCT02285816, NCT01343043, NCT03192462)^57–59^. A notable example are NY-ESO-1 targeted SPEAR T cells for synovial sarcoma, an approach that could be beneficial in DDLS given that an appropriate target is identified^60^. However, our findings indicate that commonly targeted antigens (MAGE-A3, NY-ESO-1 and SSX2) are not frequently expressed in DDLS, making them suboptimal candidate antigens. The CTAs PBK and TTK were identified to be uniformly expressed in DDLS by IHC, and significantly overexpressed compared to healthy adipose tissue by Nanostring. Additionally, we identified positively correlated patterns of PBK and TTK expression, suggesting targeting both antigens. Importantly, the success of an antigen as an immunotherapeutic target relies on its upregulation in cancer as compared to normal tissue. Evaluation of antigen expression by IHC staining revealed TTK expression in several healthy tissues, while PBK was only found to be expressed in male testes, and colon. The high expression of PBK observed in human testes tissues is negligible when considering a PBK targeted immunotherapy as germ-line cells do not express HLA complexes, and therefore PBK specific T cells would not be able to form a TCR-HLA complexes and exert their cytotoxic activity^61^. The expression of TTK in several healthy tissues may lead to negative side effects upon administration of an TTK targeting immunotherapy, thereby making PBK a potentially safer target in our study.

In addition to limited expression in normal tissue, PBK was found to be expressed in approximately 80% of WDLS samples. This is beneficial in the context of a PBK targeted therapy as DDLS is defined as a malignant neoplasm where WDLS components are often present. Overall, due to its potential contributor as an oncogenic driver, and its comparatively low expression in normal tissues, we propose PBK as a novel target antigen to develop vaccination-based immunotherapy for DDLS. To the best of our knowledge, immunotherapies targeting PBK have not yet reached the clinic. The presence of PBK specific CD8 + T cells in DDLS patient PBMCs or TILS can determine if PBK is a potent antigenic target for therapeutic vaccine approach and is a subject of further investigations. Furthermore, evaluation of cell surface expression of PBK could elucidate its potential as a viable target for TCR therapy.

In conclusion, this study revealed the existence of two distinct inflammatory phenotypes within DDLS tumors. Novel approaches that induce a broad and strong immune response against non-inflamed tumors will facilitate the development of efficient immunotherapies for DDLS. Furthermore, we identified PBK as a novel specific immunogenic target antigen in DDLS both in inflamed and non-inflamed tumors.

### Supplementary Information

Supplementary Information.

## Data Availability

The datasets generated during and/or analysed during the current study are available from the corresponding author on reasonable request.
