# Supplementary material for: Immune profiling of dedifferentiated liposarcoma and identification of novel antigens for targeted immunotherapy
Source: Sci Rep. 2024 May 16;14:11254. doi: 10.1038/s41598-024-61860-3 (PMC11099179; doi:10.1038/s41598-024-61860-3)
Supplement: Supplementary file 1 — Supplementary Information. [file 41598_2024_61860_MOESM1_ESM.pdf]

Supplementary Material

Immune profiling of dedifferentiated liposarcoma and identification of novel antigens for targeted immunotherapy

Anna Jirovec* [1,2], Ashley Flaman [3,4], Elena Godbout [2], Daniel Serrano [2], Joel Werier[4,5], Bibianna Purgina [3,4], Jean-Simon Diallo[1,2].

*** Correspondence:** Corresponding Author: ajirovec@ohri.ca

Supplemental Table 1. Individual clinical characteristics of patient FFPE samples used in nCounter Nanostring analysis

	**Immune Phenotype**	**Gender**	**Age**	**Site**	**Tumor size (cm3)**	**Primary/recurrence/metastasis**	**Treatment**	**Metastasis present at time of resection, site**	**Outcome (last visit)**
**1**	inflamed	F	70	retroperitoneal	20	primary	NA	NA	NA
**2**	inflamed	M	77	retroperitoneal	NA	primary	NA	NA	NA
**3**	inflamed	M	85	right chest wall	11	primary	Adjuvant XRT	No	deceased
**4**	inflamed	M	68	spermatic cord	NA	primary	NA	NA	NA
**5**	inflamed	M	80	left leg	15	primary	Surgery	No	deceased
**6**	inflamed	M	61	right leg	14.5	recurrence	NA	NA	NA
**7**	inflamed	F	58	left medial thigh	31	primary	Surgery	NA	NA
**8**	inflamed	F	54	left thigh	6	primary	Surgery	Yes, lungs and local recurrence	deceased
**9**	inflamed	M	75	retroperitoneal	25	primary	Neoadjuvant XRT	NA	NA
**10**	inflamed	M	71	right chest wall	5.4	primary	Surgery + Adjuvant XRT	No	disease free (2019)

**11**	inflamed	M	54	left thigh	29.6	primary	Surgery + Adjuvant XRT	No	disease free (2020)
**12**	inflamed	M	43	retroperitoneal	18.5	primary	Neoadjuvant XRT + Surgery	Yes, lung	disease free (2020)
**13**	inflamed	M	61	left medial calf	N/A	primary	Surgery	Yes, lungs	disease free (2019)
**14**	inflamed	M	71	retroperitoneal	19.7	primary	Surgery	Yes, lymph nodes	NA
**15**	inflamed	F	86	right thigh	35	primary	Surgery	No	deceased
**16**	non-inflamed	M	60	left chest wall	4.4	primary	Neoadjuvant XRT + Surgery	No	disease free (2020)
**17**	non-inflamed	F	57	left anterior thigh	10.6	recurrence	NA	NA	NA
**18**	non-inflamed	F	71	retroperitoneal	34	primary	NA	NA	NA
**19**	non-inflamed	M	47	chest wall	14.8	primary	Neoadjuvant XRT	NA	NA
**20**	non-inflamed	M	53	retroperitoneal	40	primary	NA	NA	NA

**21**	non-inflamed	F	88	left shoulder	NA	recurrence	Surgery	No	disease free (2020)
**22**	non-inflamed	M	43	left arm	1.7	recurrence	Surgery	NA	Lost to F/U
**23**	non-inflamed	F	73	right upper arm	NA	primary	Surgery + Adjuvant XRT	No	deceased, not from disease
**24**	non-inflamed	F	73	left popliteal fossa	7	primary	Adjuvant XRT	Yes, lungs	disease free (2019)
**25**	non-inflamed	M	65	retroperitoneal	31.5	primary	Surgery	No	Lost to F/U
**26**	non-inflamed	F	45	right popliteal fossa	4	primary	Neoadjuvant XRT	No	disease free
**27**	non-inflamed	NA	NA	NA	NA	NA	NA	NA	NA
**28**	non-inflamed	M	50	retroperitoneal	16.5	primary	Surgery + Adjuvant XRT + CTX	No	disease free (2018)
**29**	non-inflamed	F	75	left medial thigh	NA	primary	NA	NA	NA

NA = information not available, XRT = radiation therapy, CTX = chemotherapy

Supplemental Table 2. Differentially expressed genes in DDLS compared to healthy adipose control

Gene	Log2 Fold Change	Adjusted Benjamini- Yekutieli p-value
SAA1	-8.29	1.18E-06
CCL21	-4.22	0.00377
S100B	-4.18	3.14E-05
CD36	-4.05	1.18E-06
FOS	-3.67	1.00E-07
CXCL2	-3.5	1.50E-05
MCAM	-3.41	1.63E-12
IL18RAP	-3.35	1.18E-06
C6	-3.13	0.0018
CSF3R	-3.13	0.00394
PTGS2	-3.1	0.00158
CCL14	-3.09	4.95E-06
EGR1	-3.05	3.52E-06
ITGB4	-2.82	0.000679
CFD	-2.79	0.00215
CCL2	-2.7	0.00151
PPARG	-2.62	6.36E-05
CDH5	-2.15	1.44E-06
NLRP3	-2.07	8.92E-06
PLA2G6	-1.85	1.24E-06
ITGA	-1.71	1.13E-05
ANXA1	-1.69	5.79E-06
NOTCH1	-1.66	1.00E-07
DUSP6	-1.65	5.48E-05
PRKCD	-1.61	0.000121
STAT5B	-1.6	7.74E-06
BCL6	-1.57	0.00104
ICAM2	-1.51	0.000605
NFKBIA	-1.47	7.19E-05
CD46	-1.32	1.95E-06
BCL2	-1.19	7.19E-05
DOCK9	-1.14	0.000679
MAPK3	-0.958	0.000679
JAK2	-0.857	0.00193
TNFRSF1A	-0.659	0.00478
EP300	-0.582	0.000141
BAX	0.936	0.00158

ISG15	1.3	0.00277
GZMA	2.33	0.00343
FN1	2.44	7.19E-05
NUP107	2.86	0.0032
CDK1	2.92	3.52E-06
CD8A	2.93	0.0038
IDO1	3.07	0.00309
FCGR1A	3.1	0.00394
CXCL10	3.63	0.00104
CCR5	3.77	0.00168
BIRC5	3.86	2.19E-06
NEFL	4.23	0.000103
TTK	4.44	1.18E-06
PBK	5.17	4.41E-07

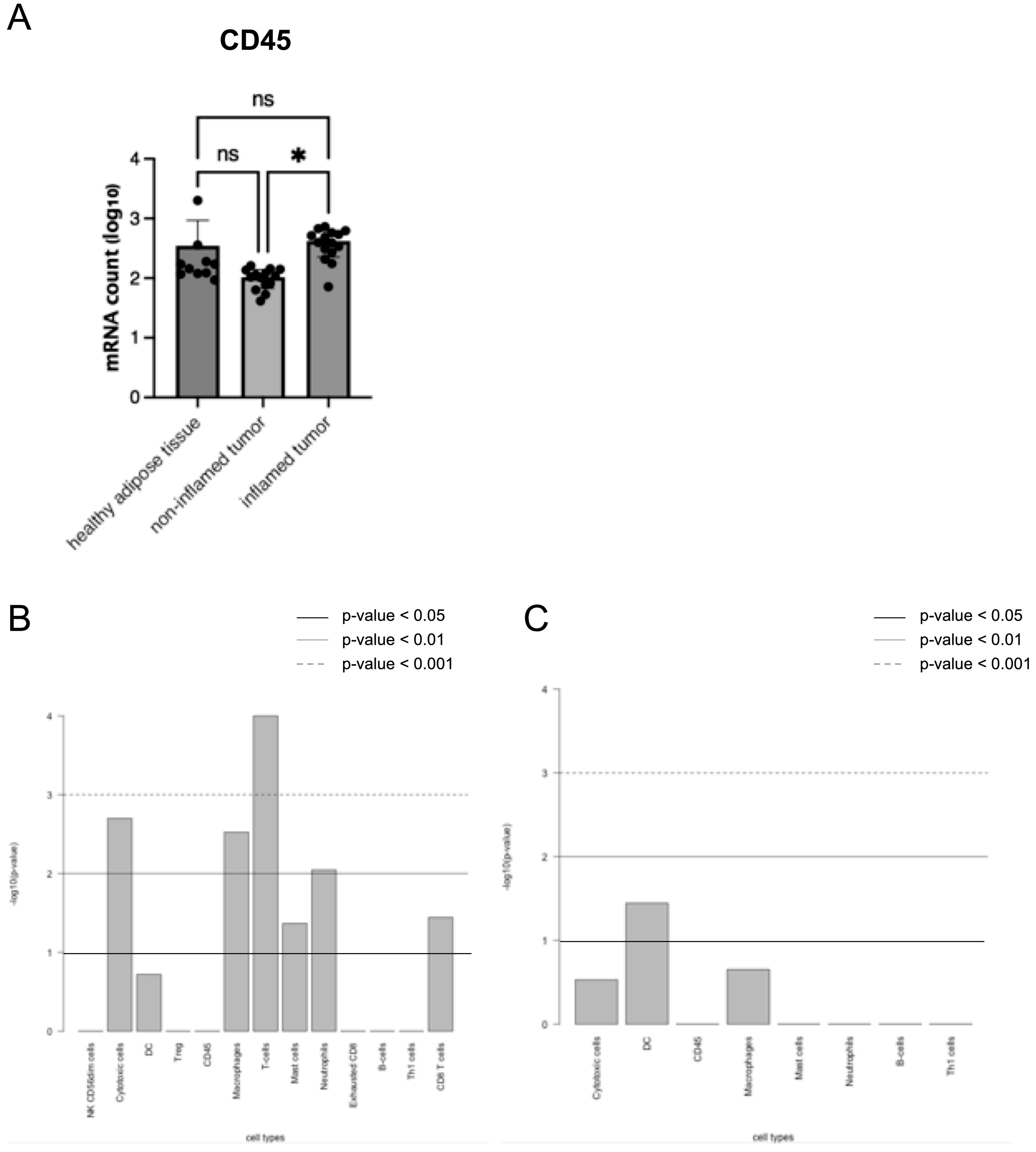

Supplemental Figure 1. **(A)** Barplot of CD45 mRNA counts in healthy adipose tissue, non-inflamed tumors, an inflamed tumors. Ordinary one way Anova, *= *p*-value < 0.05 Quality control plot exploring the validity of each cell types measurements based on detection of all pre-determined markers associated with each cell type. Barplot of p-values (-log10 transformed) across cell types in **(B)** inflamed and **(C)** non-inflamed tumors. Bars above the black line indicate cell types that were confidently detected at statistically significant levels (p-value of 0.05).

Supplemental Table 3. Table of upregulated and downregulated genes relative to healthy adipose tissue in inflamed and non-inflamed tumors, and those that are shared between both tumor phenotypes.

	**Inflamed**	**Non-inflamed**	**Shared**
**Upregulated genes**	*TRAF2, HLA-A, CD47, PSMB8, BAX, TAPBP, HLA-G, TAP1, IKBKE, HLA-B, OAS3, CSF1R, ITGA4, TAP2, HLA-DMB, CD84, C2, IL32, PSMB9, ISG15, LY96, MICB, CD74, CYBB, IL18, HLA-DRB3, HLA-DPB1, HLA-DRA, ITK, LCK, HLA-DPA1, GZMK, LY86, CCL5, IL2RB, GZMA, CD3E, IL7R, CD3D, TIGIT, CD8A, IDO1, SLAMF7, CD27, CXCL10, CXCL9, CCL17, CXCL13*	*GTF3C1, NCAM1, NUP107, NEFL*	*FN1, CDK1, BIRC5, TTK, PBK*
**Downregulated genes**	*C6, CFD, CX3CL1, DOCK9, DUSP6, EWSR1, ITGA1, ITGB4, LTF, MAPK3, NFATC1, PRKCD, PTGS2*	*BCL2L1, BST2, CCL2, CD59, CHIT1, CSF2RB, CSF3R, FOS, HCK, HLA-DMA, HLA-E, ICAM3, IL18RAP, IL1R2, ITGA1, ITGAX, ITGB4, JAK1, JAK2, LILRB3, NCF4, NFKBIA, PECAM1, PRKCD, SELL, TNFRSF14, TNFRSF1A, TNFSF12, TXNIP*	*ANXA1, BCL2, CCL14, CD36, CD46, CDH5, CXCL2, EGR1, EP300, FOS, ICAM2, MCAM, NLRP3, NOTCH1, PLA2G6, PPARG, PPBP, S100B, SAA1, STAT5B*

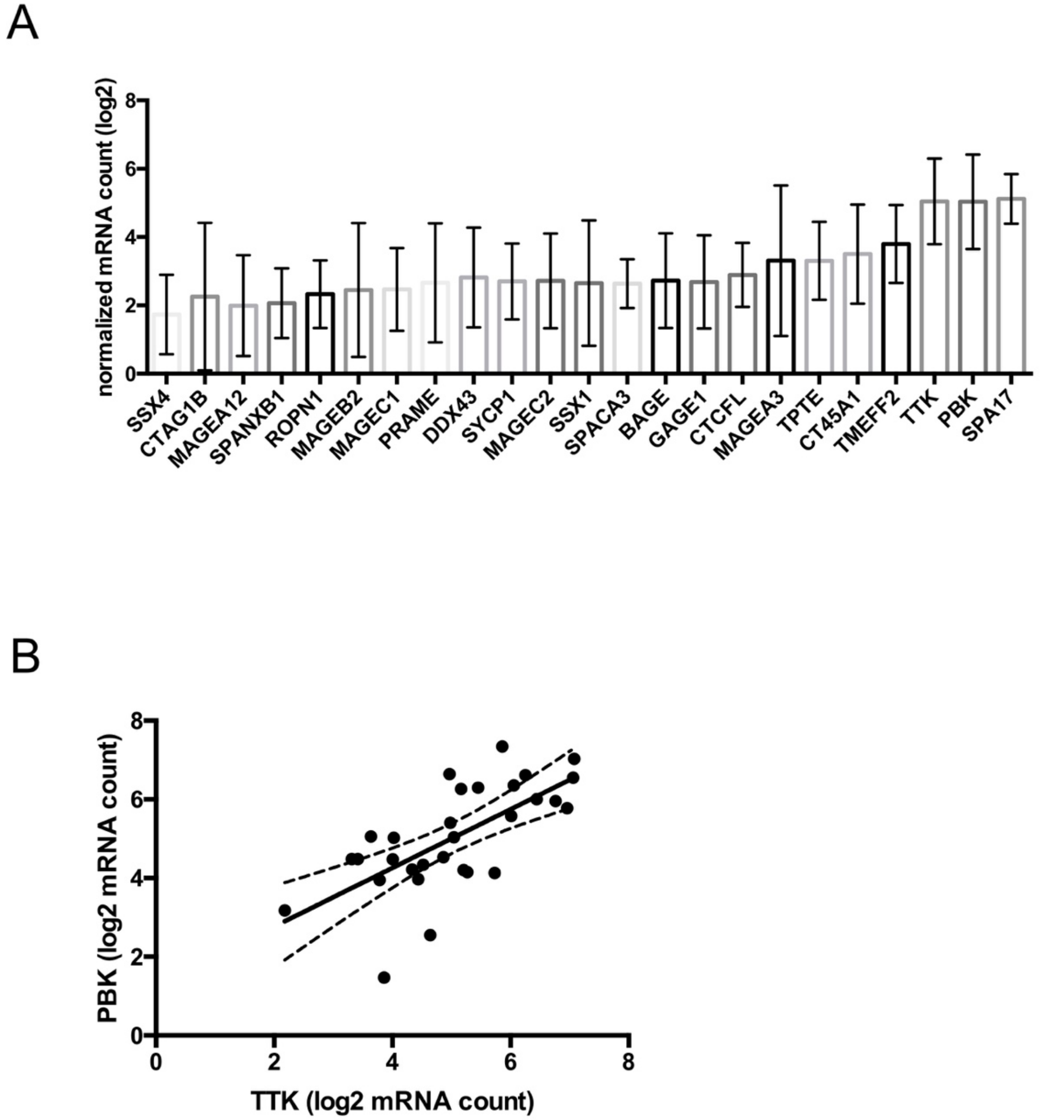

Supplemental Figure 2. (A) log2 normalized mRNA counts of CTAs in positive DDLS samples. (**B)** correlation of expression of PBK and TTK CTAs. log2 mRNA of PBK over log2 mRNA count of TTK shows a weak (r^2=0.4547) but statistically significant (p-value= < 0.0001) correlation of expression.

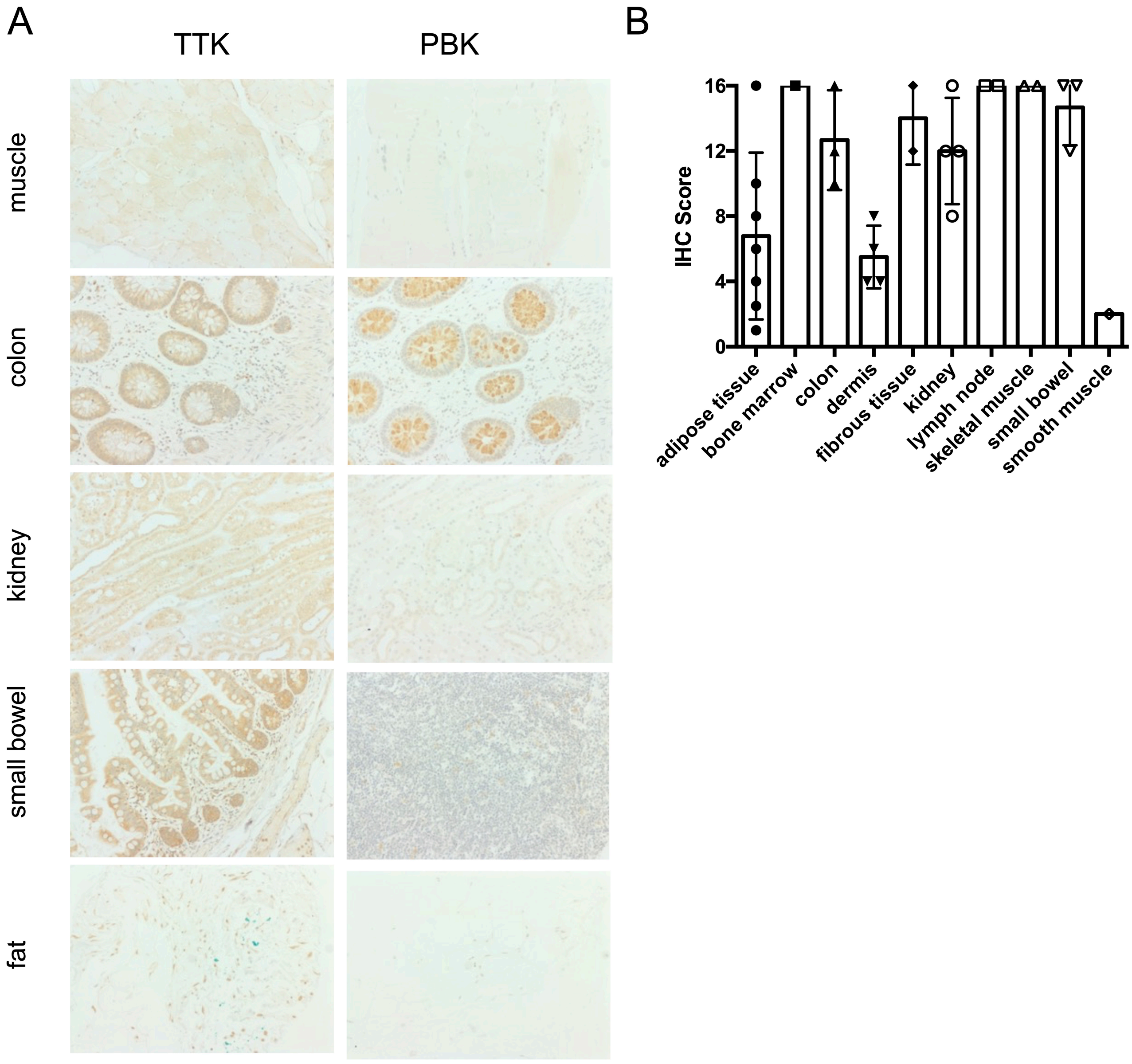

Supplemental Figure 3. (A) Representative images of positive IHC staining of TTK and PBK in in healthy tissues (**B)** IHC score of TTK expression in healthy tissues.

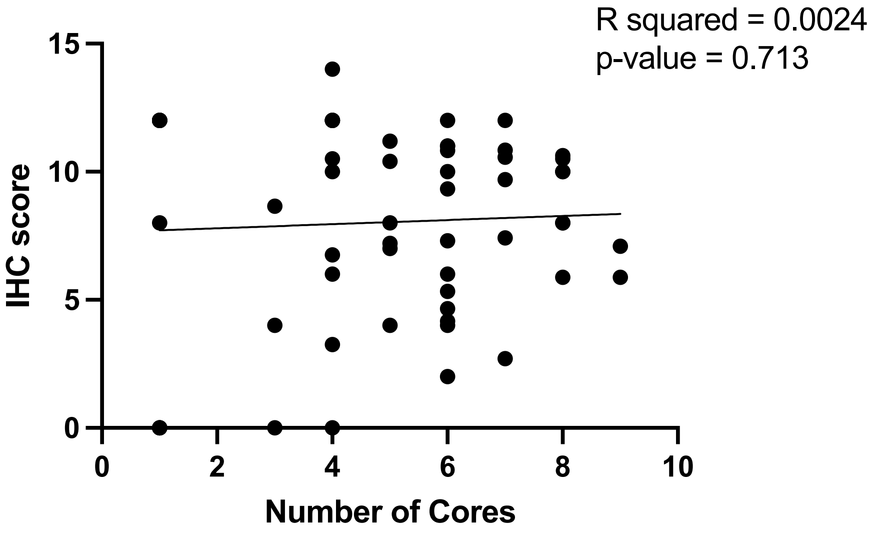

Supplemental Figure 4. Correlation analysis of IHC score and number of cores on TMA using DDLS samples stained with anti-PBK.
